# Technological advances in lower-limb tele-rehabilitation: A review of literature

**DOI:** 10.1177/20556683241259256

**Published:** 2024-06-04

**Authors:** Alireza Ettefagh, Atena Roshan Fekr

**Affiliations:** 1 KITE Research Institute, 7961 Toronto Rehabilitation Institute, University Health Network, Toronto, ON, Canada; 2 Institute of Biomedical Engineering, 7938 University of Toronto, Toronto, ON, Canada

**Keywords:** Tele-rehabilitation, lower limb disorder, remote monitoring, wearable sensors, vision-based sensors, smart mats, exercise recognition, exercise assessment, joint angle estimation

## Abstract

Tele-rehabilitation is a healthcare practice that leverages technology to provide rehabilitation services remotely to individuals in their own homes or other locations. With advancements in remote monitoring and Artificial Intelligence, automatic tele-rehabilitation systems that can measure joint angles, recognize exercises, and provide feedback based on movement analysis are being developed. Such platforms can offer valuable information to clinicians for improved care planning. However, with various methods and sensors being used, understanding their pros, cons, and performance is important. This paper reviews and compares the performance of recent vision-based, wearable, and pressure-sensing technologies used in lower limb tele-rehabilitation systems over the past 10 years (from 2014 to 2023). We selected studies that were published in English and focused on joint angle estimation, activity recognition, and exercise assessment. Vision-based approaches were the most common, accounting for 42% of studies. Wearable technology followed at approximately 37%, and pressure-sensing technology appeared in 21% of studies. Identified gaps include a lack of uniformity in reported performance metrics and evaluation methods, a need for cross-subject validation, inadequate testing with patients and older adults, restricted sets of exercises evaluated, and a scarcity of comprehensive datasets on lower limb exercises, especially those involving movements while lying down.

## Introduction

Tele-rehabilitation (Tele-rehab) is the delivery of medical or rehabilitative care to patients with rehabilitation needs using tele-communication or the internet.^1^ Tele-rehab has been available for many years, however, has had minimal implementation in clinical practice due to various reasons, such as cost, complexity, low accuracy, and high false positive rates. One major group of patients who need regular rehabilitation services are Musculoskeletal Disorder (MSD) patients. MSDs are injuries that involve muscles, tendons or nerves, ligaments, and other tissues, and are generally shown by inflammation, pain, discomfort, or tingling. They are a major cause of chronic pain, mobility impairment, falls, and loss of quality of life, creating great economic burden to the society.^2^ Lower limb MSDs known as Lower Limb Disorders (LLD) affect different parts of the lower body such as hip, thigh, knee, calf, ankle, or foot.^2^ Older adults experience a higher prevalence of LLDs due to changes in joint dynamics and muscles.

The demographic of older adults in the world population is increasing significantly.^3^ Based on the report by the United Nations, the world’s elderly population is growing faster than any other age group. It is estimated that between 2015 and 2030, the number of older adults will increase approximately 56% worldwide. In 2050, this number will reach to 2.1 billion.^4,5^ The most recent report (1 July, 2023) from Statistics Canada indicates that more than 7.5 million people in Canada are aged 65 years and older, contributing to 18.8% of the Canadian population.^6^ It is also estimated that by 2068, this number will increase up to 21.6–29.8%.^7^

MSDs rank as Canada’s third highest disease burden after cancer and cardiovascular diseases.^8^ From 1990 to 2017, all-age prevalence of MSD conditions rose from 23% to 27.8%.^8^ In 2017, Canada was among the top 10 countries globally for prevalent MSDs like osteoarthritis and gout.

A report by Safiri et al. showed that in 2017, older adults, especially women, had the highest prevalence rate of MSDs around the globe. They also highlighted that the burden due to MSDs was higher in developed countries.^9^ Musculoskeletal injuries are rarely life threatening. However, the situation may change due to possible hemorrhage.^10^ In 2020, more than 2100 casualties in Canada were caused by diseases related to the musculoskeletal system and connective tissue. This number increased by approximately 14% since 2016.^11^ Physical activity, in the form of rehabilitation programs, is recommended as a first-line treatment for long-term musculoskeletal conditions. These rehab programs are often performed under the supervision of the clinicians in clinics. However, evidence showed, that in recent years there was a drastic decline of stroke patients admitted for outpatient therapy by 50%–80%. This indicates that many patients could not receive rehab services due to social distancing barriers during the COVID-19 pandemic.^12^

It was shown that tele-rehab could represent a cost-effective approach for delivering rehabilitation services to stroke survivors, especially in cases where in-person services are not accessible. A recent study conducted in Canada provided a comprehensive cost analysis of a home-based tele-rehabilitation program specifically designed for upper limb training after stroke. This analysis includes a breakdown of various cost components associated with the program.^13^ The results indicated that providing all necessary technology (a Kinect camera, computer, monitor, etc.) costs between $475–$482 CAD per patient, while only supplying a camera would cost $242–$245 CAD per patient. These relatively low costs, in combination with tele-rehab systems eliminating travel costs for patients, supports the potential cost-effectiveness of implementing such interventions.

All these highlight the growing demand for the implementation of tele-rehab systems in clinical practice. Several studies investigated the effect of using tele-rehab systems compared to traditional rehab for different patient populations. For instance, a recent Randomized Control Trial (RCT),^14^ investigated the use of tele-rehab instead of the current standard of practice aftercare programs for patients with hip or knee replacement. The functional tests and pain assessment results from patients who used the tele-rehab program were equivalent to patients who received the current standard of practice aftercare treatments (control group). Additionally, this study found that the patients who were included in the tele-rehab program returned to work at a significantly higher rate than the control group. Another RCT^15^ studied the effect of a home-based motor training tele-rehab system in patients with stroke. The results indicated that the tele-rehab group showed significant improvement (*p = .011*) in the Fugl-Meyer Assessment compared to the conventional rehab group. Junata et al. compared the training progress of 16 patients using their proposed Rapid Movement Training (RMT) Platform to 14 patients using the Conventional Balance Training (CBT) program during 20 sessions. The training progress of the patients was quantified by Range of Motions (RoM), response times, and movement completion times during the training sessions. The study concluded that the RMT was as effective as the CBT, providing beneficial effects on post-stroke patients.^16^ Similarly, Gandolfi et al. proposed a home-based balance training program using Virtual Reality (VR) and a Nintendo Wii Fit (Nintendo, Kyoto, Japan) for Parkinson Disease (PD) patients. In this study, 76 patients were randomly assigned to receive either in-home proposed tele-rehab (*n* = 38) or in-clinic Sensory Integration Balance Training (SIBT).^17^ The results demonstrated a significant improvement on the Berg Balance Scale in the patient group undergoing VR tele-rehab (*p* = .04).^18^ In a study by Tao et al., the effectiveness of the WiiNWalk intervention in improving walking-related physical functions and balance confidence in older adults with lower limb amputation was investigated. An RCT was designed with a total of 71 lower limb prosthesis users, who performed Wii Fit modified activities for balance training.^19^ The efficacy of the intervention was evaluated using a 2-min walk test. The study’s results revealed that while WiiNWalk did not improve walking-related physical functions, it did enhance users’ balance confidence.^20^

In addition, several studies investigated the usability analysis of the tele-rehab systems to understand how well the patients can learn and use the proposed platforms to achieve their care goals. For example, Oyama et al. conducted a pilot study to test the efficacy of their tele-rehab system using a markerless system (Simi Motion Analysis, Simi Reality Motion Systems, Germany) in combination with an insole pressure sensor (eRubber shoes, Toyoda-Gosei, Japan). They recruited 7 participants with a history of hospitalization to perform physical therapy tasks in a clinic. A clinician from another hospital supervised the patients remotely during the performance of the rehab tasks. The information such as participants’ posture, walking speed, joint positions, and the pressure heat maps of the foot, along with the video of the participants performing the exercises were monitored by the remote clinician in real-time. The system was evaluated by a qualitative questionnaire. According to the results, most of the participants expressed a preference for remote rehabilitation. Initially, only two participants preferred on-site rehabilitation, but their preference shifted to “I would rather engage in remote rehabilitation” after the experiment.^21^

Furthermore, Medina et al., studied the usability of their platform called ePHoRt with 39 participants.^22^ They used the IBM Computer System Usability Questionnaire to determine the subjective evaluation of participants and their self-reported feedback. Their results suggest that ePHoRt was effective, and easy to use. Additionally, their main findings show that “*user guidance is a critical aspect to ensure a good usability of the tele-rehab platform*.”^22^ Palestra et al. proposed a rehab system to prevent falls among older adults.^3^ The usability of their system was evaluated based on data from 6 senior participants. Their system’s protocol consisted of 5 exercises with three levels of difficulty, primarily focusing on lower limb exercises. Their results showed a significant improvement in maintaining a physical activity among older adults. Additionally, they reported that the performance of the postural response was increased by 80% on average. Another study in^23^ conducted two User Experience (UX) evaluations based on behavioral observations and questionnaires. The results from this study showed an increase in motivation of patients toward game-based therapy which combines telemedicine with gaming technology to make rehabilitation fun and engaging. It was also mentioned that the clinicians found the developed mini-games practical, since the gaming sessions could be customized based on each patient’s needs.

Moffet et al. developed a home-based tele-rehab platform called eChez-Soi. They recruited 4 patients to participate in a tele-rehab program. The program consisted of upper and lower extremity strengthening exercises and cardiovascular training. Patient data was collected through various devices including a wrist-worn pulse oximeter, a wearable gait analysis system (LegSys, Biosensics), Wii Fit, and the Xbox Dance Mat. The results from the study indicated an overall satisfaction rate of 4.63 ± 0.43 among all users.^24^ Another pilot study by Coats et al. also supported the feasibility of this program.^25^ They recruited 5 patients with similar inclusion criteria and assessed the functional capacity of patients using the 6-min walking test (6MWT) and timed stair test (TST). The results showed significant improvements in TST and 6MWT after the tele-rehab program (3.0 ± 0.19 s, *p* = .05 and 39.8 ± 19.7 m, *p* = .01, respectively). These findings suggest that the eChez-Soi platform has the potential to maintain or improve the functional capacity of patients.

Several studies have sought to use different kinds of technologies in designing their tele-rehab platforms. In this paper, we focus on reviewing recent studies in this regard, comparing the performance of different modules used in tele-rehab. We identified and discussed the existing gaps in these studies for possible further research. The rest of the paper is organized as follows: The Methods section outlines how the literature search was conducted. The Results section presents recent literature in the field of tele-rehab systems and the associated technologies. The Discussion section reviews and summarizes the common methods of the research presented in the Results section and explores future directions. The Conclusion section wraps up the review.

## Methods

The literature search was conducted from November 2022 to August 2023, and only the studies published in English were included. Google Scholar and the University of Toronto’s Online Library databases were the main search engines used for this review paper. Databases such as IEEExplorer, PubMed, and Semantic Scholar were also searched for papers. The term “tele-rehab” was always searched as “tele-rehabilitation/tele-rehabilitation.” Likewise, “rom” was searched as “range of motion.” The following is a list of key search terms used, where the ‘+’ signifies “and” and the ‘/’ signifies “or”: tele-rehab, tele-rehab + lower limbs/extremities, tele-rehab + ankle/hip/knee, tele-rehab + feedback/biofeedback, tele-rehab + feedback/biofeedback + lower, tele-rehab + assessment/evaluation/monitoring, tele-rehab + feedback/biofeedback + realtime, tele-rehab + machine learning/deep learning, tele-rehab + rom, tele-rehab + rom + lower, tele-rehab + vision + lower, tele-rehab + pressure/pressure mat/smart mat + lower, tele-rehab + IMU/wearables + lower, tele-rehab + Kinect/xtion/depth camera/camera, tele-rehab/rehab + recognition/assessment/evaluation/monitoring + depth/pressure.

In total, we examined more than 100 papers in the field of tele-rehab, selecting approximately 70 relevant papers that met our inclusion criteria. Our inclusion criteria for this review were: (1) studies that focused on tele-rehabilitation technologies for joint angle estimation, activity recognition, and exercise assessment, (2) published between 2014 and 2023, and (3) manuscripts written in English. Studies focusing on literature reviews, theses, conference abstracts, and books were excluded. Additionally, given the technological focus of our review, we excluded studies related to usability factors and comparisons between tele-rehab and conventional rehab, such as RCTs.

**Figure 1.**
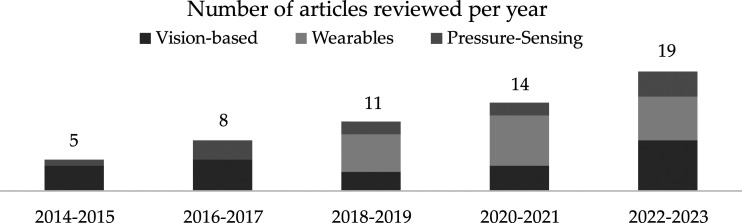
Number of articles we reviewed correlating to each year.

Out of the 70 papers that were reviewed, 57 used different kinds of technologies toward developing a home-based tele-rehab platform. The remaining studies, as discussed in Section 1, centered on either usability analysis or comparing tele-rehab with conventional rehab programs, such as, RCTs. Among the subset of 57 papers, 24 papers used vision-based, 21 used wearable, and 12 used pressure-sensing technologies. Since our literature review was focused on lower-limb tele-rehab, most of the studies concentrated on upper-limb tele-rehab were excluded from our results. However, some studies that examined both upper-limb and lower-limb exercises remained included. Nevertheless, this inclusion of upper-limb studies was inevitable because the ideas and methods employed in lower- and upper-limb tele-rehab are intertwined. Since this is a state-of-the-art review paper, the focus of literature was from the past 10 years, as shown in Figure 1.

## Results

To address home-based rehabilitation programs, a variety of systems have been designed with different technologies such as vision including depth and RGB cameras,^26–49^ and wearables, such as Inertial Measurement Units (IMU).^50–69^ Some systems also used pressure-sensing technologies, such as pressure sensitive mats (with electronic textiles) and insole pressure sensors,^70–81^ to measure the pressure of body limbs and its distribution on the respective areas. In each subsection, we begin with the outcomes of studies that performed joint angle measurement or estimation, followed by the studies that performed exercise recognition and/or exercise quality assessment. Finally, a brief summary is provided after each subsection. Section 4 discusses the findings in more detail.

### Vision-based technology in tele-rehab

Vision-based techniques are one of the popular approaches in designing tele-rehab platforms. This approach mostly uses 2D (e.g., RGB) or 3D (e.g., Kinect) cameras and computer vision algorithms, mainly skeleton tracking models, to extract joint information from the captured data. This section will review 24 papers using vision-based approaches in designing automatic tele-rehab systems. Existing studies applied these methodologies for joint angle/RoM estimation,^26–29,38,40,42–46,48^ exercise recognition,^27,30–32,35,41^ exercise evaluation, and biofeedback system via providing quality scores or visual cues.^27,31,33–37,39,41,45–47^

#### Reviewed studies

KINOPTIM was a tele-monitoring platform developed to reduce the risk of falls among older adults in home environment.^29,38^ The proposed system remotely monitors users through a series of virtual reality (VR) games that were set by clinicians. The data was collected from a depth camera (Asus Xtion), and two wearable motion sensors (accelerometer and gyroscope) to evaluate the position of the body, and active RoM of limbs. The OpenNI library^82^ was used to identify the body joints using the RGB and depth data. The system helps clinicians decide whether to use proactive or active interventions.^29,38^ In another study, Zhao et al. proposed a home-based tele-rehab system using Kinect and a rule-based framework to assess the quality and quantity of performed exercise.^45^ They recruited 8 healthy participants to perform 3 rehab exercises including bowling, hip abduction, and sit to stand. RoM and joint angles were measured by the Kinect system. A clinician defined certain thresholds for each exercise. According to these thresholds, the system provides users with a real-time visual feedback within its avatar-based guidance system.^46^ The authors in^28^ proposed a tele-rehab system called KiReS using Kinect. They tested their system with 7 total hip replacement patients. In this study, the patients were asked to perform 10 lower-limb rehab exercises such as hip flexion, abduction, adduction, flexion, extension, etc. A clinician set appropriate exercise parameters in the system for each patient. Patients were provided with visual feedback through a 3D avatar, allowing them to observe their own movements and receive performance feedback in comparison to the physiotherapist’s avatar. Based on the findings, patients experienced a significant improvement in their exercise capabilities as measured by their system.^27^ Similarly, Adolf et al.^26^ used a single RGB camera with OpenPose^83^ skeleton tracker to measure joint angles during 6 rehab exercises. Data were collected from 5 normal participants and a professional. Measurements from the professional determined the average RoM for different working limbs in each exercise. Their system records the exercise videos from users and directly streams them to a remote server. The videos get processed in the server and the clinician can remotely select each exercise and visualize the joint angles of working limbs along with the RoMs defined by the professional. They claimed that the physiotherapist can make a basic assessment just by taking a glance at the generated graphs. Albeit useful, these studies did not provide any results on system validation versus any gold standard to make sure the RoMs are estimated with adequate accuracy.

Rybarczyk et al. introduced a web-based system for the remote monitoring of rehab exercises, called ePHoRt.^42^ Similar to KiReS, this platform uses Kinect to record patients’ movements. To evaluate the Kinect system’s precision in measuring arm angles compared to an accelerometer sensor as their gold standard, 4 participants were recruited to perform shoulder abduction exercises. The results showed a significant correlation between both systems in terms of Pearson’s coefficient of correlation (*r* = 0.96 ± 0.03). Ye et al. proposed a marker-based Motion Capture (Mocap) system using a single depth camera (Kinect 2).^43^ They measured 14 participants’ (5 healthy, and 9 stroke survivors) kinematics parameters such as knee joint angles, velocity, movement patterns, gate cycle, step, and stride length, as well as swing and stance duration to monitor their progress of clinical therapy. Gait events were detected with a maximum mean error of 1.75% compared to manually-labeled ground truth. Knee joint angles from 5 subjects were captured while walking. The results were compared to VICON^84^ mocap system. The maximum Root Mean Squared Error (RMSE) with the proposed system was under 6°.

Similar to the previous work, a recent research study employed a stationary Light Detection and Ranging (LiDAR) depth camera along with Cubemos and Mediapipe skeleton trackers for the estimation of joint positions.^48^ The method’s validity was confirmed by comparing its results to data obtained from a Mocap system. 13 participants were involved in the assessment of a shoulder abduction exercise. The initial findings of the study emphasize the effectiveness of Cubemos over Mediapipe, as it produces a Mean Absolute Error (MAE) of less than 10°. However, it is worth noting that the proposed system has limitations when it comes to capturing movements directed towards the camera.

A new version of the Kinect Software Development Kit (SDK) 2 skeleton tracking system was proposed by Yu et al. to measure the RoM of body limbs in tele-rehab.^44^ This study focused on the upper and lower arm joints. Data was collected from 6 participants. The authors compared their estimated RoMs to other motion tracking systems such as Kinect v1, Kinect v2, and a marker-based optical tracking system called Polaris Vicra.^85^ Their results showed that the proposed system could remove the systematic error of Kinect skeleton tracking algorithms, particularly at full flexion. Additionally, the estimated RoMs from their system provided a mean error of 3.78° compared to the clinical gold standard goniometer. Rosique et al. presented an augmented reality mirror called ExerCam, that used vision-based human pose detection based on OpenPose. The main objective of this system was to provide a web application with two interfaces, one for patients and one for clinicians to monitor the patients and change or modify the plan of care, remotely. The data was collected from an RGB camera with an augmented virtual skeleton superimposed on the patient’s body. A preliminary study was conducted with 20 participants to evaluate the precision of RoM measurement. The exercises were shoulder abduction, elbow flexion, hip abduction, and knee flexion. Each exercise was repeated 3 times. The average estimated RoMs were achieved with less than 3% relative error compared to measurements from goniometer as their ground truth.^40^

In addition to joint angle/RoM measurement, several tele-rehab platforms focused on exercise recognition, assessment and quality scoring using vision-based technologies. For example, Anton et al. developed a system to identify physical rehabilitation exercises and assess their correctness in real time using Kinect.^27^ Their algorithm incorporated two methods: (1) Posture classification, and (2) Exercise recognition with trajectory recognition. The system used the spatial coordinates of body joints captured by Kinect to calculate relative positions, angles between joints, and angles between limbs. These measurements were then used to construct a posture descriptor consisting of 30 features to reduce the data dimensionality. Posture classification was achieved by comparing the captured descriptor with pre-stored posture descriptors using Dynamic Time Warping (DTW). For exercise recognition, the system identified the initial and final postures of each exercise. It then used a trajectory recognition method based on DTW to identify and assess the correctness of movement patterns between the initial and final postures. To evaluate the proposed algorithm, clinical trials were conducted with 15 patients with shoulder disorders, yielding an exercise assessment accuracy of 95.16% for the binary classification of the correctness in 6 different shoulder exercises. Barriga et al. presented a vision-based system for telecare and tele-rehab using a depth camera (Asus Xtion), and neural networks.^30^ They claimed that the proposed system can automatically classify 7 static postures and falls. They validated their system with data from 6 participants. In addition, they investigated different parameters such as number of hidden neurons, maximum error, learning rate and learning function in their neural network design, which was based on Multi-Layer Perceptron (MLP). In addition, they investigated the impact of the distance from the camera, as well as angle between the camera and subjects (for skeleton tracking system). They could achieve the best accuracy of 96% for static postures and fall classification.

Another Kinect-based tele-rehab platform was proposed by Rybarczyk et al.^41^ for patients after hip replacement surgery. They developed an AI module based on DTW and HMM to assess the correctness of the movements performed by the users in real time. The inputs to the AI module were the joint angles, and coordinates calculated by Kinect system. A graphical user interface was designed to provide users with feedback within and after each trial. To validate the DTW method, they recruited 7 healthy participants to perform hip abduction, hip flexion, knee flexion, and hip extension. Participants were instructed to introduce a different error in the execution of each exercise at each repetition. Each trial was assessed by four physiotherapists and classified as good, or bad movement. The performance of the DTW algorithm was validated against the clinician’s assessments. The accuracy of the algorithm was 88%. Furthermore, to validate the HMM method, they used data from 4 healthy participants. The only rehab exercise was a sequence consisted of doing one step forward, one step sideways and one step backward. Each participant repeated this exercise for 70 times in 5 different correct ways and one incorrect way, resulting 6 different HMM models. Each model reached 100% accuracy except for one model which had 57%. Thus, the average accuracy was 92.83%.

Barzegar Khanghah et al. introduced an automated vision-based system that leverages ML techniques to classify the correctness of 9 different rehabilitation exercises.^49^ Their models were trained using the 10 features that were extracted from 24 different joint angle signals acquired from the skeleton data of an available online dataset named IntelliRehab Dataset (IRDS).^86^ This dataset consisted of data from 16 patients and 14 healthy participants performing 9 exercises. Data were videos recorded from a Kinect 1 sensor and included a binary performance correctness label. Their study explored the effectiveness of 6 different ML-based classifiers, including Random Forest (RF), MLP, NB, Support Vector Machine (SVM), K-Nearest Neighbors (KNN), and Logistic Regression. The results of the study indicated an average accuracy of 89.86% and an F1-Score of 72.84% for the 10-Fold cross-validation method. In the LOSO cross-validation, the average accuracy was 88.21%, with an F1-Score of 68.16%.

Guo and Khan^36^ proposed a rehab exercise assessment method using OpenPose and RGB cameras. They also used the KIMORE^87^ dataset to evaluate their method. They trained different ML models such as SVM, RF, and k-Nearest Neighbors (kNN) to predict the performance scores of each exercise in the KIMORE dataset. The input of their models was features extracted from 2D skeletal joints from the RGB videos. Their results show an average Spearman’s rank correlation coefficient of 0.522 for all 5 exercises. Decroos et al. developed an ML pipeline using Kinect to monitor and assess the correctness of physiotherapy exercises performed by patients at home.^35^ Their pipeline involved three main steps: identifying individual exercise repetitions, representing time-series data with statistical features about joint angles, and detecting the exercise’s type, correctness, and possible mistakes using ML. To evaluate the performance of their method, they recorded 10 healthy participants performing 3 rehab exercises (squats, forward lunges, and side lunges) while tracking joint movements with Kinect. The participants were also instructed to perform incorrect ways of performing exercises further for a mistake detection task. For exercise recognition, they used five learners including Logistic Regression, NB, Decision Trees (DT), RF, and XGBoost. The input feature vector to the learners consisted of 150 summary statistics (30 angles × 5 statistics - min, max, mean, median, std). The best accuracy achieved was 99% using XGBoost algorithm with Leave-One-Subject-Out (LOSO) cross-validation. In addition, in the task of classifying exercises into correct and incorrect categories, the XGBoost algorithm achieved an accuracy rate of 73.4%. Additionally, when detecting mistakes within incorrectly performed exercises, the algorithm demonstrated a classification accuracy of 73.8% for identifying three different types of mistakes.

The use of deep learning models, especially Long-Short-Term Memory (LSTM) networks and Graph Convolutional Networks (GCN) has become popular in the fields of activity recognition and exercise quality assessment, particularly in the tele-rehab. These AI models offer unique advantages for capturing complex patterns and relationships in data, making them valuable tools for these applications. For instance, a home-based rehab system called Tele-EvalNet was proposed by Kanade et al.,^37^ which consists of live-feedback, and movement quality assessment modules. The live-feedback module used Kinect data to generate visual feedback. This feedback was based on the dissimilarity measure (euclidian distance) between patient’s data and the expert’s stored template. Furthermore, they proposed a CNN-LSTM model to predict patients’ movement quality score. They evaluated their model’s effectiveness on the KIMORE dataset that provides performance scores between 0 and 50 for five rehab exercises performed by 44 healthy subjects and 34 patients. Performance scores predicted by their model deviated from KIMORE’s scores with an average RMSE of 0.13012 after rescaling all the scores to [0, 1].

The presence of 3D Convolutional Neural Networks (CNN) is also observed in tele-rehab applications. Namely, Barzegar Khanghah et al. proposed a vision-based biofeedback system to assess the correctness of rehab exercises in IRDS, this time using deep learning techniques.^31^ First, they used a pre-trained 3D CNN to identify each exercise. Average accuracies of 96.62% ± 0.88% and 86.04% ± 0.14% were achieved using 10-Fold and LOSO cross validation, respectively. Furthermore, to assess the correctness of the exercises, they labeled each correctly identified exercise to “*Correctly executed*,” and each misclassified exercise as “*Incorrectly executed*,” thereby forming a binary exercise assessment model. Using this approach, they obtained average accuracies of 90.57% and 83.78% using 10-Fold and LOSO cross validations, respectively.

Bijalwan et al. proposed a heterogeneous deep learning model to identify lower-limb rehab exercises.^32^ They considered a total of 10 exercises involving abduction, flexion, rotation, and dorsi-flexion of the lower limb on both the left and right sides. These exercises were performed by 25 healthy and 10 crouch walking subjects. Depth data was collected from a Kinect v2 sensor. They employed CNN and CNN-LSTM models to classify these exercises. Their experimental results demonstrated equal accuracy and F1-score of 96% for the CNN model and 98% for the CNN-LSTM model using hold-out cross validation. Chowdhury et al.^33^ also proposed two LSTM networks to predict exercise quality scores. The first model incorporates predefined features in the KIMORE dataset, while the second model involves feature extraction via a GCN applied to the skeletal data. Their results indicated that the LSTM model performs better when using features extracted by the GCN. Predicted exercise scores deviated from the ground truth scores with RMSE values of 0.29 and 0.19 for each model, respectively. Similarly, a Spatial-Temporal Graph Convolutional Network (ST-GCN) was used by Deb et al. to predict continuous scores for the evaluation of physical rehabilitation exercises.^34^ Their method introduced an LSTM module within the ST-GCN architecture to capture temporal features from exercises of varying lengths in order to enhance the network’s adaptability for regression tasks. Additionally, they proposed a self-attention mechanism using ConvLSTM layers to assess the importance of individual body-joints in predicting the final assessment score. This highlighted the joints that had the highest contribution in predicting the assessment score for the users. Their model was evaluated on KIMORE and University of Illinois Physical Rehabilitation Movement (UI-PRMD)^88^ datasets, and demonstrated average MAE of 0.01 for 10 exercises in the UI-PRMD dataset and 0.58 for five exercises in the KIMORE dataset.

Zheng et al. introduced a rotation-invariant skeleton-based system for assessing rehab exercises.^47^ They employed 4 RGB cameras and a pre-trained model for human pose estimation to extract skeleton joints. Their dataset included 529 push-up samples collected from 16 healthy participants. Each sample was labeled as correctly or incorrectly executed based on a standard of correctness that focused on maintaining a straight trunk line, proper elbow angle, and avoiding collapsed waist or extended neck. Their deep learning framework was based on ST-GCN and achieved 85.44% accuracy through a hold-out subject-based validation in classifying push-up correctness. Moreover, using a 3:1 split hold-out validation, they achieved average accuracies of 97.41%, 98.8%, and 98.96% for classifying exercises in IRDS, UI-PRMD (Kinect part), and UI-PRMD (VICON part) datasets, respectively. Furthermore, the authors integrated Gradient Class Activation Mapping (Grad-CAM) into their GCN-based assessment model. They established a mapping function to highlight incorrectly positioned joints in red and correct ones in blue based on CAM values, as a means of providing visual feedback to the users.

Graph transformers also hold a great promise in rehabilitation exercise assessment. In a recent study, Réby et al. proposed a deep learning model based on this approach for the assessment of physical rehabilitation exercises.^39^ They evaluated their model using the mocap data from the UI-PRMD dataset, which consists of data from 10 healthy subjects performing 10 rehabilitation exercises including deep squat, hurdle step, inline lunge, side lunge, sit to stand and standing active straight leg raise exercises. The predicted exercise quality scores deviated from the ground truth with MAE and RMSE values of 0.0167 and 0.0143, respectively. Additionally, for the binary classification of detecting correct and incorrect exercises, the model achieved an F1-Score of 85%.

#### Summary

The reviewed studies predominantly used RGB or depth cameras along with computer vision and skeleton tracking algorithms for joint angle/RoM estimation, exercise recognition, and exercise quality evaluation in vision-based tele-rehab systems. Several studies reported accurate joint angle and temporal gait parameter estimation when validated against motion capture or goniometer measurements, with errors generally under 4° for angles. For exercise recognition and correctness assessment, techniques such as DTW, HMMs, and ML models achieved high accuracies, typically between 85% and 90%. Deep learning approaches using CNNs, LSTMs, and GCNs further improved performance, reaching over 95% accuracy for exercise classification and RMSEs under 0.2 for predicting continuous quality scores on benchmark datasets. These systems often provided real-time visual feedback to users based on extracted joint data and employed techniques, such as Grad-CAM, for highlighting incorrect body positioning. Table 1 summarizes the reviewed studies that used vision-based technology in tele-rehab.

**Table 1.** Summary of vision-based studies.

Ref.	# Sub^a^	# Post/Ex^b^	Technology	Main objective	Performance
^45,46^	8 healthy	3	Kinect	Development of a tele-rehab platform	—
^26^	5	6	1 RGB	Development of a tele-rehab platform	—
^40^	20 patients	4	1 RGB	Development of a tele-rehab application	—
^28^	7 patients	10	Kinect	Development of a tele-rehab platform	—
^42^	4	1	Kinect	Joint angle measurement	*r* = 0.96 ± 0.03
^48^	13	2	Depth camera	Joint angle estimation	MAE: 10°
^44^	6 healthy	2	Kinect 1, Kinect 2, polaris Vicra, Kinect 2+	RoM measurement	Mean error: 18.7° Mean error: 6.5° Mean error: 8.37° Mean error: 3.78°
^43^	5 healthy	1 (gait)	Kinect	Development of a mocap	RMSE: 6°
^30^	6	7 (static)	Depth camera	Posture classification	Accuracy: 96.00%
^27^	15 patients	6	Kinect	Exercise assessment	Accuracy: 95.16%
^32^	25 healthy, 10 patients	10	Kinect	Exercise recognition	Accuracy: 98% F1-score: 98%
^41^	7 healthy for DTW 4 healthy for HMM	4 + 1	Kinect	Exercise assessment	Accuracy: 88% Accuracy: 92.83%
^31^	14 healthy, 16 patients (IRDS)	9	Kinect	Exercise assessment	Accuracy: 90.57%
^31^	14 healthy, 16 patients (IRDS)	9	Kinect	Exercise recognition	Accuracy: 96.62%
^49^	14 healthy, 16 patients (IRDS)	9	Kinect	Exercise assessment	Accuracy: 89.86%
^37^	44 healthy, 34 patients (KIMORE)	5	Kinect	Exercise assessment	RMSE = 0.13012
^36^	44 healthy, 34 patients (KIMORE)	5	Kinect	Exercise assessment	Mean *r* = 0.522
^35^	10 healthy	3	Kinect	Exercise recognition Exercise assessment Mistake detection	Accuracy: 99% Accuracy: 73.4% Accuracy: 73.8%
^47^	16 healthy	1	4 RGB	Exercise assessment	Accuracy: 85.44%
^34^	10 healthy (UI-PRMD) 44 healthy, 34 patients (KIMORE)	10	Kinect Mocap	Exercise assessment	MAE: 0.0114 MAE: 0.582
^33^	44 healthy, 34 patients (KIMORE)	5	Kinect	Exercise assessment	RMSE: 0.192
^39^	10 healthy (UI-PRMD)	5	Mocap	Exercise assessment	MAE: 0.0167 RMSE: 0.0143 F1-score: 85%

^a^Number of participants enrolled in the study, categorized as healthy (able-bodied individual) or patients. Absence of a designation indicates missing information in the reviewed study.

^b^Number of postures or rehabilitation exercises considered in the study.

### Wearable technology in tele-rehab

Several wearable techniques are proposed to design tele-rehab platforms. These wearables are either in the form of smart textile,^50–53^ Surface Electromyography (sEMG)^54–58^ or wearable sensors such as IMUs.^59–65,67–69^ This section will review 21 papers using wearable body sensors. Similar to the vision-based approach, the reviewed studies either estimated joint angles/RoMs or used ML for activity recognition. In both applications, some studies performed exercise quality assessment by using either threshold-based approach or ML for classifying the correctly and incorrectly executed motion patterns.

#### Reviewed studies

##### Textile technology

Haladjian et al. designed a smart textile bandage for knee named KneeHapp. The hardware involved two motion sensors and an electric circuit made of elastic conductive threads. The system used threshold-based and peak detection algorithms to provide live feedback to the patients while exercising. These exercises include one-leg squats and hops, and side-hops. Their system also estimated the RoM. To validate results, they collected data from 10 patients and measured four different angles of flexion for each participant’s leg (80 in total). To obtain the ground truth, they aligned straight markers along participants’ upper and lower leg and calculated the angle between the markers on 2D photographs. An average error of 4.82° ± 3.92° was reported when measuring the flexion.^52^

Wood et al. developed 3 knee sleeve devices equipped with 16 piezoresistive sensors to calculate knee joint angles for at-home knee rehabilitation.^51^ To assess the accuracy of their device, they recruited 18 healthy participants to perform open-chain knee flexion exercises. They used 18 ML regressors to predict joint angles. The accuracy of the models was evaluated using RMSE and R-Squared metrics, and the best model was selected for joint angle prediction. The subject-specific models showed an average RMSE of 7.6° and 1.8° for flexion/extension and internal/external rotation, respectively, while the device-specific models showed an average RMSE of 12.6° and 3.5° for flexion/extension and internal/external rotation, respectively. Similarly, Nakamoto et al. proposed a method for measuring knee and ankle joint angles using a stretchable strain sensor attached to the skin.^53^ The sensor measured the stretching length of the skin around a joint during knee flexion/extension and ankle dorsiflexion/plantarflexion exercises. They used a linear regression model to calculate the joint angles using the output voltage of the strain sensor. The method was tested on one healthy participant. According to the results, the average error between the estimated angles and the reference joint angles measured by a mocap system was 4.4° ± 3.6° for knee, and 1.9±°1.7° for ankle. The correlation of determination was 0.98.

Davarzani et al. developed a wearable smart sock prototype to track ankle joint angle during gait movement.^50^ The sock was equipped with four stretch-based and one pressure-based soft robotic sensor (SRS) to capture data. They trained multivariable linear regression and two deep learning models, LSTM and CNN networks, to estimate foot joint angles in sagittal and frontal planes and validated them using an optical mocap system. Subject-specific models were created for 10 healthy subjects walking on a treadmill, and the prototype was tested at various walking speeds to assess its ability to track movements for multiple speeds and generalize models for estimating joint angles. The LSTM model was found to be the most accurate, with lower MAE, lower RMSE, and higher R-squared values. The average MAEs were 1.138° and 0.939°, and RMSEs were 1.46° and 1.11° in sagittal and frontal planes, respectively.

##### SEMG sensors

SEMG was found to be used in different applications of tele-rehab systems including exercise monitoring and biofeedback (gamification),^56,57^ exercise classification^55^ and joint angle/RoM estimation.^54,58^ For example, an sEMG-based biofeedback system was proposed by Yassin et al.^57^ Their platform enabled therapists to remotely monitor and control their patients’ rehabilitation programs based on sEMG signals. Their system consisted of a low-cost sEMG data acquisition device placed on the biceps muscle, as well as two smartphone applications – one for the patient and one for the therapist. The patient application displayed three different biofeedback visualizations, including a car game, gauge indicator, and bar graph, which were designed to provide visual feedback on sEMG signal amplitude. The maximum amplitude of each contraction was recorded for the clinician. To evaluate the system’s effectiveness, sEMG signals were collected from five participants lifting different weights and compared to signals gathered from a BIOPAC certified device^89^ as the ground truth. The signals from the proposed system provided a strong correlation of 95.5% with the ground truth. Additionally, there was a 90.89% similarity in their Root Mean Square (RMS) values.

Marin-Pardo et al. proposed a muscle-computer tele-rehab system called REINVENT, which uses sEMG signals.^56^ Their system captured sEMG signals from the extensor and flexor muscles of the wrist, processed the signals, and provided visual feedback through two games. The authors calculated the Extension Rate, defined as the ratio of the extensor muscle to the sum of extensor and flexor muscles, as a metric to evaluate the system’s performance. The system was tested on 2 patient participants for 40 rehabilitation sessions, and the results showed a significant increase in the Extension Rate (*p < .001*). The authors suggested incorporating control thresholds into the system to encourage participants to perform more exercises.

Wang et al. aimed to classify trunk movements with and without spinal orthosis.^55^ 10 healthy participants were evaluated to identify movements using sEMG signals in this study. Two modalities were used to test participants: motion without the spinal orthosis (Normal) and with the spinal orthosis (Spinal orthosis). Surface electrodes collected sEMG signals from eight muscles during four movements (flexion-extension, lateral bending, axial rotation, and stand to sit to stand). Three ML algorithms -RF, k-NN, and SVM – were compared for their classification performance. The results showed that the classification accuracies for distinguishing between Normal and Spinal orthosis movements were 94.24%, 97.12%, and 98.56%, respectively. Additionally, the classification accuracies for identifying the four different movements in the Normal modality were 92.86%, 95.71%, and 91.43% for RF, k-NN, and SVM, respectively. For the Spinal orthosis modality, the classification accuracies were 85.71%, 88.57%, and 91.43% for RF, k-NN, and SVM, respectively.

Wang et al. aimed to predict lower limb joint angles for lower limb rehabilitation.^58^ To achieve this goal, they introduced an sEMG feature extraction technique based on wavelet packet decomposition. They also developed a prediction model using an Extreme Learning Machine (ELM). They recruited a total of 12 healthy individuals to perform rehabilitation tasks in six different scenarios that included slow and fast knee extension and flexion, walking, and squatting. Five channels of sEMG signals were recorded. Their results indicated that their model achieved an accuracy of 96.23% ± 2.36% via 6-Fold cross validation in predicting the joint angles.

A new framework for online prediction of joint angles was introduced by Song et al. using an LSTM neural network.^54^ Their research involved the collection of sEMG, joint angle, and plantar pressure data from five healthy participants. They collected sEMG data from eight leg muscles. They recorded a total of six different joint angles, which included measurements from the right hip, knee, and ankles. These data were captured using a 12-camera mocap system equipped with 15 markers. Additionally, they collected plantar pressure signals while the subjects walked on a level treadmill designed to detect exerted forces. They employed two input configurations for their network, including the use of only sEMG (unimodal) and a combination of sEMG with plantar pressure data (multimodal). Following online feature extraction and standardization, these inputs were used to train the LSTM-based angle prediction model. According to the multimodal setting outcomes, the mean values of RMSE, MAE, and Pearson correlation coefficient for the three joint angles were within the ranges of [1.33°, 2.84°], [1.06°, 2.07°], and [0.975, 0.9958], respectively.

##### Inertial sensors

Inertial Measurement Units (IMUs) are electronic devices that measure and report a body’s angular rate and orientation, using a combination of accelerometers, gyroscopes, and sometimes magnetometers. IMUs are commonly used in tele-rehab systems to measure joint angles.^59,60,62,65,68,69^ Majumder et al. designed a sensor fusion algorithm to estimate the joint angles of thigh, knee, and ankle using two IMUs attached above and below the joints. They validated the performance of their algorithm versus outputs from mocap system. The data from three healthy volunteers who walked on a treadmill at four different speeds was used. Their estimates of joint angles had a RMS deviation of 2.5° and average Pearson correlation coefficient of *r* = 0.951.^63^ Argent et al. evaluated the performance of various ML algorithms, including Linear Regression (LR), Polynomial Regression (PR), DT, and RF, for estimating hip and knee joint angles using a single IMU.^67^ The accuracy of the algorithms was measured by comparing the estimated angles with the ground truth data obtained from a 3D CODA Mocap system. 14 healthy participants were recruited to perform eight rehabilitation exercises. The RF algorithm achieved the best result with an average RMSE of 4.81° ± 1.89°.

In addition to measuring the joint angles and RoMs, plenty of studies focused on rehabilitation activity recognition and biofeedback systems.^59,60,69^ This application could help clinicians understand the activities performed by the patients on a daily basis. For instance, a wearable device called PhysioSens^59^ was developed for real-time monitoring of three lower-limb exercises: knee extension, held hip flexion, and hip abduction and adduction. This device was equipped with a 3-axis accelerometer and a microcontroller, and was placed near the ankle. A set of 33 features (11 for each accelerometer axis) was extracted from accelerometer signal and used as input to a dense neural network with two hidden layers. They achieved an F1-Score of 99% and an accuracy of 99.41% using a hold-out cross validation. The authors, however, did not provide any details regarding their dataset. Lai et al. proposed a personalized rehab system for lower-limb exercise recognition using an IMU attached on the knee and instep.^62^ Two subjects were recruited and asked to perform six lower limb exercises. SVM and Adaptive Neuro-Fuzzy Inference System (ANFIS) models were trained by 63 extracted features. Using 5-Fold Cross-validation, the best accuracy they achieved in classifying different exercises was 99%.

García-de-Villa et al.^60^ used 4 IMUs to recognize and assess exercises. They used six classifiers in three distinct methods: (i) a single classifier for exercise recognition and assessment, (ii) exclusion of incorrect movements for exercise recognition only, and (iii) a two-stage approach involving recognition followed by assessment. They recruited 30 healthy participants to perform eight rehab exercises, five of which were lower-limb exercises. ML classifiers used were SVM, DT, RF, KNN, ELM, and MLP. The first method achieved an accuracy of 88.4% and an F1-Score of 88.8% using RF. For the second approach, SVM yielded the highest accuracy of 91.4% and F1-Score of 92.6%. In the third method, SVM resulted in the highest F1-Score of 96.4% and accuracy of 96.2% for exercise recognition, and the highest F1-Score of 96.66% and an average accuracy of 97.15% for exercise assessment. Reported performance were based on LOSO. Moreover, Bevilacqua et al. used motion signal captured from an IMU sensor located on the midpoint of the sheen of about 50 healthy and 20 patient participants to assess the correctness of four physical movements including heel slide, seated knee extension, inner range quadriceps and straight leg raise.^65,68^ They used LSTM networks to identify temporal patterns of the time series. The pointwise predictions of each physical movement were then aggregated using different boosting methods. Using LOSO cross-validation, the best average binary (correct and incorrect) classification accuracy was 92.32% in the healthy test set, and 79.19% in the clinical test set.

Pereira et al. proposed a new method for evaluating the quality of rehabilitation exercises using both inertial sensors and a sEMG-based exercise tracking system.^69^ To evaluate their approach, they recruited 17 patients and tested their methodology to assess the correctness of two specific exercises: isometric scapular retraction strengthening (exercise 1) and forward lunge (exercise 2). Inertial sensors were attached to the wrists for exercise 1, and on the thigh and ankle for exercise 2 to monitor movements. Each repetition of the exercises was annotated by physiotherapists as correct or incorrect. The ML pipeline was divided into two stages: automatic segmentation of repetitions based on sEMG and supervised ML to classify repetitions into correct or incorrect executions. Using k-NN, SVM, and RF, the classification accuracies for exercise 1 were 97%, 95.5%, and 98.5%, respectively. Also, for exercise 2, the classification accuracies were 95.5%, 95%, and 96.5%, respectively.

IMU signals are also used in diagnosis applications.^61,64^ Abdollahi et al. proposed a sensor-based ML model to classify patients with Nonspecific Low Back Pain (NLBP) into different risk categories.^64^ They recruited 94 patients who attached an IMU sensor on their sternum and performed trunk flexion and extension movements in the sagittal plane. The collected data was processed, and 19 different features were extracted. The SVM and MLP classifiers were employed, and the results suggested accuracy rates of 75% and 60%, F1-Scores of 74.6% and 63.7%, and recalls of 72.5% and 66.2% using SVM and MLP, respectively. The scores from the clinical subjective StarT Back Screening Tool (SBST)^66^ were used as the true labels for the classifications. Kim et al. conducted a study to detect Sarcopenia patients using gait parameters obtained by IMUs attached to the right and left foot.^61^ They recruited 10 sarcopenia and 10 normal participants and recorded IMU data from the sensors. The study extracted spatial-temporal parameters used in clinical practice and descriptive statistical parameters for all 7 gait phases from the data. Feature selection was performed using the Shapley Additive method. Classification methods, including SVM, RF, MLP, and deep learning methods, were employed to identify sarcopenia. The results indicated that the knowledge-based gait parameter detection method was more accurate in identifying sarcopenia than automatic feature selection using deep learning. The SVM model achieved the highest accuracy of 95% using LOSO, and with 20 descriptive statistical parameters.

#### Summary

The reviewed studies explored various wearable technologies like smart textiles, sEMG sensors, and IMUs for joint angle/RoM estimation, exercise recognition, and quality assessment in tele-rehab systems. Smart textile devices achieved average errors around 4–7° for knee joint angle measurement when validated against motion capture. SEMG signals combined with machine learning models like extreme learning machines could predict lower limb joint angles with over 96% accuracy. For exercise recognition using sEMG, classifiers like SVMs and RFs reached up to 98% accuracy. IMU-based systems reported RMS errors around 2.5–4.8° for joint angle estimation compared to motion capture, and up to 99% accuracy for exercise classification using neural networks and fuzzy inference models. ML models trained on IMU and sEMG data achieved 92–98% accuracy in assessing exercise quality and correctness. Wearables were also explored for diagnosis of conditions like low back pain and sarcopenia, with SVMs proving effective at around 75–95% accuracy. Table 2 summarizes the reviewed studies that used wearable technology in tele-rehab.

**Table 2.** Summary of studies which used wearable technology.

Ref.	# Sub	# Post/Ex	Technology	Main objective	Performance
^52^	10 patients	4	Textile	Joint angle measurement	Mean error: 4.82°± 3.92°
^51^	18 healthy	2	Textile – 16 piezo-electric sensors	Joint angle measurement	RMSE (flexion/extension): 7.6° RMSE (internal/external): 1.8°
^53^	2 healthy	1	Textile – 1 strain sensor	Joint angle measurement	MAE: 4.4° ± 3.6° MAE: 1.9° ± 1.7° *r* = 98%
^50^	10 healthy	1	Textile – 5 sensors	Joint angle estimation	MAE: 1.14°, RMSE: 1.46° MAE: 0.94°, RMSE: 1.11°
^58^	12 healthy	6	sEMG	Joint angle estimation	Accuracy: 96.23% ± 2.36%
^54^	5 healthy	6	sEMG and plantar pressure	Joint angle estimation	RMSE: 1.63° MAE: 1.27°
^63^	10	4	2 IMUs	Joint angle measurement	RMSE: 2.5°
^67^	14 healthy	8	1 IMU	Joint angle measurement	RMSE: 4.81° ± 1.89°
^57^	5 healthy	-	sEMG	Biofeedback	*r =* 95.48%
^56^	2 patients	-	sEMG	Biofeedback	—
^55^	10 healthy	4	sEMG	Exercise recognition	Accuracy: 98.56%
^59^	—	3	1 IMU	Exercise recognition	Accuracy: 99.41% F1-score: 99%
^65,68^	50 Healthy, 20 patients	4	1 IMU	Exercise recognition	Accuracy: 92.32% Accuracy: 79.19%
^62^	2 healthy	6	1 IMU	Exercise recognition	Accuracy: 99%
^60^	30 healthy	8	4 IMUs	Exercise recognition+assessment	Accuracy: 96.2% Accuracy: 97.15%
^69^	17 patients	2	3 IMUs	Exercise assessment	Accuracy: 97.50%
^64^	94 patients	1	1 IMU	Risk level categorization	Accuracy: 75%
^61^	10 healthy, 10 patients	Gait	2 IMUs	Sarcopenia detection	Accuracy: 95%

### Pressure-sensing technology in tele-rehab

Pressure sensitive mats and insoles are used to recognize patterns of movement such as posture, limb alignment, and center of balance. This technology provides pressure heatmaps of the body (or feet) and allows researchers to analyze the pressure distribution of areas that are in contact with the body. In this section, we review 12 papers that used this technology in tele-rehab. Some of these papers used this technology in combination with other technologies such as vision or wearables. Similar to the previous approaches, the reviewed studies either estimated joint angles, or gait parameters^71,75,79^ or used ML for activity recognition.^70,73,74,76,77,80,81^ However, it is worth noting that there is still a noticeable lack of work in the area of exercise assessment modules using this technology, as only one study was identified and categorized within this domain.^72^

#### Reviewed studies

Bayan et al. proposed an experimental rehab system for patients with lower-limb injuries.^78^ Their system was made of VR eyeglasses for providing a virtual environment, EMG shield for analyzing muscle activity, an accelerometer for tracking the movements of users, and pressure sensors to measure the feet forces on the ground. sEMG and accelerometer sensors were attached on participants’ feet. Data were collected from 10 candidates: 5 healthy and 5 patients with rheumatism. A clinician specified a gaming profile for the user depending on their situation. Their results showed an enhancement of the amplitude of EMG in users over time, suggesting the development of stronger muscles. Ultimately, they observed different Force Sensitive Resistor (FSR) profiles between the two participant groups. No quantitative results were reported in this study.

Choffin et al. developed a method to predict lower body joint angles by utilizing plantar pressures in shoes.^71^ The study involved 37 participants performing squats while wearing a custom-designed footwear sensor comprising of six force-sensing resistors and a microcontroller to aid in joint angle prediction. To validate the accuracy of the results, a wearable mocap system (Xsens) was employed to measure 3D joint angles as the ground truth. Using the Gaussian Process Regression algorithm, the researchers created a progressive model that predicted the angles of ankle, knee, hip, and lumbosacral joint. Notably, the lumbosacral joint angle during the testing phase was predicted with an RMSE of 0.3°, based on data from one randomly selected participant. The overall RMSE of all predicted joint angles ranged from 0.21° to 3.29° across the *X*-axis and 0.22° to 3.77° for the *Y*-axis. These results were obtained from testing with two randomly chosen participants.

Martini et al. developed a pressure-sensitive insole system for the estimation of temporal gait parameters in real time using optoelectronic sensors and threshold-based algorithms.^75^ The system was assessed against a commercial force plate recording the vertical component of the ground reaction force (vGRF) and the coordinate of the center of pressure along the antero-posterior plane CoP_AP_ in 10 healthy participants during ground-level walking at two speeds. The results indicated a Median Absolute Error (MedAE) of 0.06 s and Interquartile Range (IQR) of 0.02 s, and 0.04 (0.02) s for heel-strike and toe-off recognition, respectively. The insoles were able to estimate the stance phase duration with a MedAE of 2.02 (IQR = 2.03)% and showed a MedAE Pearson correlation coefficient of 0.96 (IQR = 0.02) with force platform for CoP_AP_ profiles.

Jagos et al. developed a research project named eSHOE, which is a gait analysis system.^79^ The overall goal of this project was home-based monitoring and training for people suffering from chronic diseases which affect their locomotor system. They used motion and insole pressure sensors to collect movement data directly from users’ feet. Data was collected from 11 hip fracture patients and 12 healthy participants. They extracted different gait parameters from the motion signals and validated their outputs against GAITRite platform which is a gold standard reference in gait analysis. The average differences ranged from −0.046 to 0.045 s in the patient group, and −0.029 to 0.029 s in the healthy group.

Bennett et al. aimed to analyze the trajectory of sit-to-stand-to-sit movements in individuals with and without stroke.^70^ The researchers used a full body mocap suit with 17 IMUs to obtain the ground truth for the movements. They also used a balance board with four pressure sensors to measure the center of force and a pressure sensing mat to analyze weight distribution. ML models were created using parameters such as height, weight, BMI, and age. The models used weighted kNN and linear regression to predict trajectories. Results showed that the predicted trajectories in non-stroke subjects matched the true trajectories with an average R-squared of 0.864 (SD = 0.134). However, stroke patients exhibited larger within-class variation, indicating the need for larger-scale trials to obtain significant results.

A new textile technology referred to as 3DKnITS^73^ was presented by Wicaksono et al., which incorporates a specific sensing textile mat and a deep learning model. They aimed to classify 7 basic activities and 7 yoga poses in real-time. The deep learning approach employed in this study was based on personalized CNN models. The researchers used pressure heatmap data collected from a single healthy subject, and hold-out validation to assess the performance of their model. They reported accuracies of 99.6% and 98.7% in classifying basic activities and yoga poses, respectively.

Huang et al. proposed a framework to detect and monitor five on-bed rehab exercises.^81^ They collected data using a pressure sensitive bedsheet from 10 participants. Their method consisted of preprocessing pressure heatmaps, dimensionality reduction with manifold learning, and activity recognition using manifold matching. According to their results, the best classification precision and recall were 90.34% and 90.14% respectively.

Similarly, Sun et al. developed an recognition system to identify six on-bed rehab exercises.^80^ They collected pressure map data from 15 participants. In their method, pressure maps were divided into three regions: upper (head region), middle (torso and limb region), and lower (leg and foot region) and further subdivided into limb clusters using K-means. The centroid for each region was calculated and selected as centers for kNN to perform a limb matching algorithm. According to the results, their system achieved an overall accuracy of 97.8%. No cross validation method was reported by the authors.

Wijekoon et al. proposed Multi-modal Exercises Dataset (Mex)^76^ as a multi-sensor Human Activity Recognition (HAR) dataset. Data was collected with a pressure mat at 15 Hz, a depth camera at 15 Hz, and two accelerometers at 100 Hz. The first accelerometer was placed on the thigh, and the second was on the wrist. The dataset includes lower-limb exercises (7 exercises in total) and consists of data from 30 healthy participants. The authors also presented the benchmark performance of their dataset in the exercise recognition task, using different ML and deep learning methods. The average F1-Score for classification of different exercises using Leave-Multiple-Subjects-Out (LMSO) cross-validation was 86.34%, 88.92%, 64.99% and 71.945% for depth, accelerometer (on the thigh), accelerometer (on the wrist), and pressure data, respectively. They concluded that visual data like depth and pressure data were better represented with CNNs, whereas time-series data from accelerometers were better with Discrete Cosine Transform (DCT) and trained with LSTM. Furthermore, the same authors proposed a multi-modal Hybrid Attention Fusion (mHAF) architecture in.^74^ They achieved an F1-Score of 96.24% using LOSO cross-validation for classification of different exercises in their proposed dataset with combination of pressure mat, depth camera and accelerometer (placed on the thigh) data. They claimed that mHAF learns feature importance and modality combinations for different exercise classes.

Lee et al. proposed a gait type classification method based on deep learning using a smart insole with various sensor arrays.^77^ Gait data was measured using a pressure sensor array consisting of eight pressure sensors, an acceleration, and a gyroscope sensor. Features of gait pattern were then extracted using a deep CNN. In order to accomplish this, measurement data of continuous gait cycle were divided into steps. A feature map was then extracted by constructing an independent deep CNN for data obtained from each sensor array. Each of the feature maps were then combined to form a fully connected network for gait type classification. Experimental results for 7 types of gaits (walking, fast walking, running, stair climbing, stair descending, hill climbing, and hill descending) from 14 participants showed that the proposed method provided an accuracy of more than 90% using 7-Fold cross-validation.

Wang et al. presented a gait monitoring method to recognize patients with Knee Osteoarthritis (OA) by assessing the plantar pressure signals during walking.^72^ Pressure signals were collected by a smart insole embedded with a flexible piezoresistive array (4 × 12 matrix). Their experiment involved 18 participants diagnosed with Knee OA and 22 control participants. The plantar surface was divided into eight areas to calculate the contact time and maximum force of each area in a gait cycle. These characteristics were used to train an SVM, which achieved an accuracy of 93.15%, a precision of 92.39%, and a recall of 92.79% using a 7:3 subject-based split validation.

#### Summary

The reviewed studies used pressure sensitive mats and insoles for joint angle/gait parameter estimation, as well as activity recognition in tele-rehab systems. Some studies estimated joint angles for example, lumbosacral angle with RMSE under 0.3° or temporal gait parameters for example, stance phase duration with median errors around 2% when validated against motion capture or force plates. For activity recognition, pressure data was often fused with vision or wearable sensors. Deep learning models achieved high accuracies around 97–99% for classifying exercises, gait types, and activities using multi-modal data including pressure sensing. Only one study focused specifically on exercise quality assessment for knee osteoarthritis patients using insole pressure data, reporting 93% accuracy. Table 3 summarizes the reviewed studies that used pressure-sensing technology in tele-rehab.

**Table 3.** Summary of reviewed studies which incorporated pressure-sensing technology.

Ref.	# Sub	# Post/Ex	Technology	Main objective	Performance
^78^	5 healthy, 5 patients	NA	VR + IR + pressure sensors + EMG shield	Development of a multi-sensor rehab system	—
^71^	37	1	Footwear sensor	Joint angle estimation	RMSE: [0.21°, 3.29°] in *X*-axis RMSE: [0.22°, 3.77°] in *Y*-axis
^75^	10 healthy	Gait	Smart insole	Gait parameter estimation	MedAE: 0.06 ± 0.02 s MedAE: 0.04 ± 0.02 s MedAE *r* = 0.96 ± 0.2
^79^	12 healthy, 11 patients	NA	1 IMU + Insole pressure sensor	Gait analysis	[−0.046, 0.045] s (patient) [−0.029, 0.029] s (healthy)
^77^	14	Gait	Smart insole	Exercise recognition	Accuracy: 90%
^73^	1 healthy	14	Pressure mat	Activity recognition	Accuracy: 99.15%
^81^	10	5	Pressure mat	Exercise recognition	F1-score: 90.24%
^80^	15 healthy	6	Pressure mat	Activity recognition	Accuracy: 97.8%
^76^	30 healthy	7	1 depth camera 1 IMU on thigh 1 IMU on wrist Pressure mat	Exercise recognition	F1-score: 78.20% F1-score: 90.15% F1-score: 63.15% F1-score: 74.05%
^74^	30 healthy	7	1 depth camera + 1 IMU + pressure mat	Exercise recognition	F1-score: 96.24%
^72^	18 healthy, 22 patients	Gait	Smart insole	Exercise assessment	Accuracy: 93.15%

## Discussion

In our analysis, we have identified several key modules essential for the development of an effective automatic tele-rehab platform. These modules include but are not limited to joint angle estimation, exercise recognition, and exercise assessment, which may involve correctness prediction, exercise scoring, or (bio)feedback.

### Joint angle estimation

**Figure 2.**
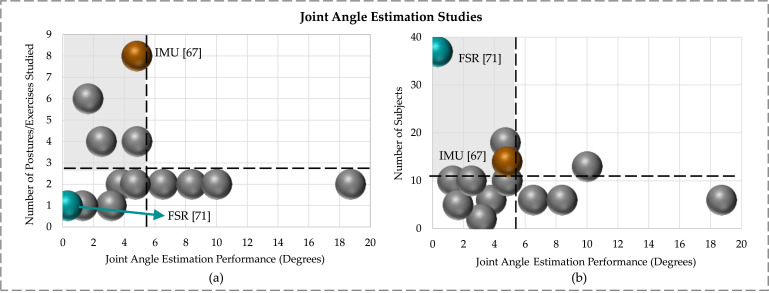
Comparison of joint angle measurement/estimation performance (average errors) between studies with respect to (a) the number of postures/exercises, and (b) the number of subjects included.

Joint angle estimation plays a pivotal role in tele-rehab as they enable monitoring and assessment of patient movements and RoMs. In the reviewed studies, we observed a range of methods and techniques for this goal. For example, vision-based technologies such as RGB cameras coupled with 2D skeleton tracking modules (e.g., OpenPose) were used to estimate the RoMs. Additionally, RGBD cameras equipped with skeleton tracking modules, such as the widely favored Kinect, were also prevalent in these investigations. Asus Xtion, paired with the OpenNI SDK, represented another viable option in this category. In studies involving wearable technology, a range of sensors were used, including motion sensors, strain sensors, stretch sensors, sEMG, and IMUs. These sensors provided data for the development of different ML and deep learning regression models to estimate joint angles. In the reviewed studies involving pressure-sensing technologies, only one study was found to estimate joint angles using data captured by force-sensing resistors. Figure 2 shows the reviewed studies that reported joint angle estimation performance in terms of RMSE, MAE and errors in degrees. In the reviewed studies, Argent et al. considered the maximum number of postures (14) for joint angle estimation using 14 participants,^67^ as shown in Figure 2(a) and (b) with brown dots. Conversely, Choffin et al. used the highest number of subjects for joint angle estimation by integrating six wearables, focusing on just 1 exercise type,^71^ shown in Figure 2(a) and (b).

These figures indicate that on average, the reviewed studies considered approximately three postures and 10 subjects, as illustrated by the dashed lines in Figure 2(a) and (b), respectively. The grey-shaded region in the figure indicates the area of interest, representing small errors and a relatively large number of subjects and postures/exercises considered. One challenge in comparing different studies was that they used different types of ground truths for joint angle measurements. Most studies relied on Mocap systems, including wearable systems like Xsens and optical systems like VICON, to establish their reference standards. An alternative approach was observed in one study, which used calculated joint angles from an accelerometer as their ground truth.^42^ Moreover, the use of classical goniometers was observed in two studies.^40,44^ It is important to note that goniometer measurements could potentially lead to variability due to observer judgment, as indicated by prior research.^90^ Finally, one study used reference angles derived from markers within 2D photographs.^52^

### Exercise recognition and quality assessment

Automatic recognition and assessment of exercises performed by patients is another fundamental aspect of tele-rehab. Automatic exercise recognition enhances patient autonomy and allows them to adjust their routine in a dynamic way.^35^ It could also provide the patient with a daily/weekly/monthly summary of performed exercises, tracks the progress made in the prescribed exercise routine by the physiotherapist. This could offer valuable information for the clinicians. Furthermore, an exercise recognition model can also be integrated into an exercise assessment model, as shown in.^31^

The studies we reviewed demonstrated a wide array of methodologies employed in this field. From traditional rule-based systems and template-matching methods, such as DTW, to more techniques, such as probabilistic methods like HMM, and ML classifiers, such as RF and SVM, which provided the highest performance metrics compared to other ML methods. It is, however, important to note that the performance of such methods heavily rely on the quality of the features used as a representation of data.

In addition to traditional ML methods, deep learning models are showing great promise in this field. Unlike ML, features are learned throughout the training process in these models. Five noteworthy methods in this category include CNNs, LSTM networks, and graph-based models such as GCN, ST-GCN, and Graph Transformers.

CNNs can capture spatial relationships in images and are capable of recognizing exercises from visual data. LSTMs can capture temporal aspects of exercise movements. Graph-based models excel in learning exercise patterns by analyzing the movement of joints, that is, key points extracted from pose estimation models or motion capture systems. One notable advantage of graph-based methods is their ability to provide visual feedback by highlighting the joints involved in incorrect exercise execution.^34,47^ It is, however, essential to consider the computational demands and resource requirements associated with these deep learning approaches, especially when deploying them in resource-constrained environments. Zheng et al. distinguished two branches within vision-based assessment models: image-based and skeleton-based. Image-based models, referred to as large models, aim to extract motion features directly from images for example, RGB or depth. In contrast, skeleton-based models, referred to as small models, first identify the human skeleton using devices like Kinect or pre-trained pose estimation models, and then train the assessment model with this skeleton data. It has been claimed that skeleton-based methods are favored due to their simplicity in model training.^47^

**Figure 3.**
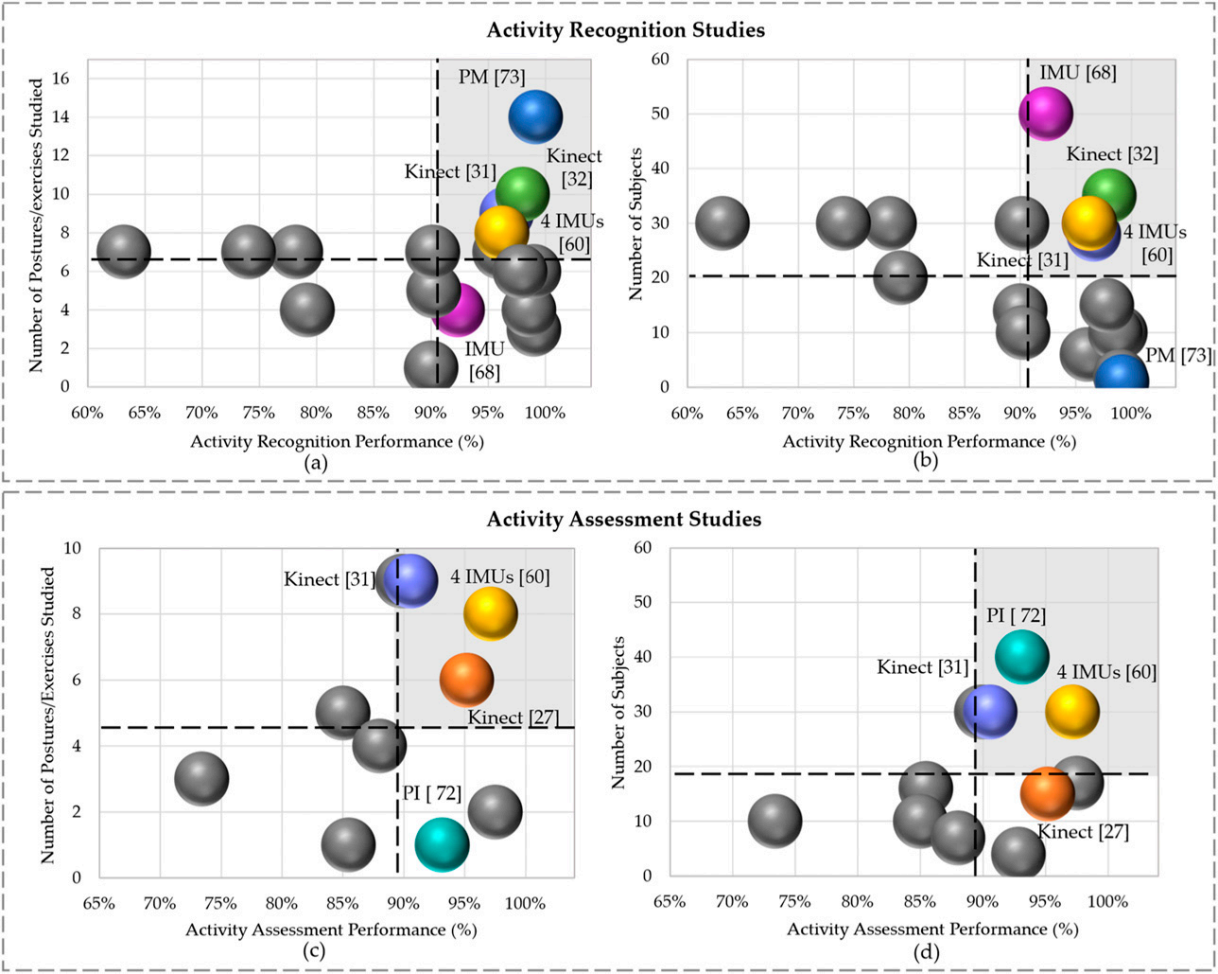
Comparison of activity classification performance between studies with respect to (a) the number of postures/exercises and (b) number of subjects. Comparison of assessment performance between studies with respect to (c) the number of postures/exercises and (d) number of subjects.

Vision-based technologies were found to be a more popular approach in various aspects of tele-rehab. Compared to the wearable technology, vision-based is a more convenient approach for the primary users of tele-rehab, the older adults and patients, as their exercise movements would not get influenced by the worn devices.^47^ In addition, research has shown that the use of wearable devices in a real-life scenario is questionable, as the older adults are not keen to wear those devices.^91^ Pressure-sensing technology remains underexplored in the realm of tele-rehab. This technology offers unique advantages, particularly in mitigating the occlusion problem that exists in vision-based approaches.^92^ This issue is even more likely to happen during performing lower-limb exercises. However, it is important to note that pressure data has considerably less spatial resolution when compared to data obtained by cameras. By combining data from pressure sensors with vision devices, the overall performance of recognition and assessment modules could be improved.^74,93^ Figure 3 shows all reviewed papers that focused on activity recognition and assessment with different technologies, subject sample sizes, and postures/exercises. The dashed lines in this figure represent the average values of performance, number of subjects and postures/exercises over all reviewed studies. For instance, Figure 3(b) shows that, on average, all studies depicted considered 20 subjects, with an approximate action recognition performance of 91%.

Wicaksono et al. (blue dot in Figure 3(a) and (b)) achieved a maximal recognition performance of 99.15% with the largest number of classes (14), but only with 1 subject (the smallest set).^73^ This indicates that pressure data could be effective for recognizing activities with limited number of subjects and a relatively larger set of classes. Considering the grey shaded areas in this Figure 3 as a region of interest with high performance and larger number of subjects and postures, Bijalwan et al., García-de-Villa et al., and Barzegar Khanghah et al. obtained a recognition performance of 98%, 96.2% and 96.6%, with 10 classes and 35 subjects, and eight classes and 30 subjects, and 9 classes and 30 subjects, respectively.^31,32,60^ This shows that reaching a high recognition performance along with considerable amounts of subjects and postures is feasible using Kinect or multiple IMUs on the body. Bevilacqua et al. reported a recognition performance of 92.32% with four classes (below average) and 50 subjects (maximum).^68^

As shown in the region of interest of Figure 3(c) and (d), Barzegar Khanghah et al. and García-de-Villa et al. achieved an assessment performance of 90.57% and 97.15%, with 9 and 8 classes, respectively,^31,60^ and both had equal sample size of 30 subjects. Wang et al. attained an assessment performance of 93.15% using data from pressure insoles with 40 subjects (maximum) and only 1 class (walking).^72^ The authors in Anton et al. reported a high assessment performance of 95.16%, with a relatively large number of classes (6), and 15 subjects, notably, all were patients.^27^

According to Figure 3, it can be observed that study^60^ appears in all regions of interests, indicating that it evaluated a large number of postures and subjects, delivering high performance in both action recognition and assessment. However, using 4 IMUs on the body may not be practical or comfortable for an automated tele-rehab system, especially for older adults. Given this, the Kinect and pressure-sensing technology stand out as promising options for a more user-friendly and feasible tele-rehab approach.

### Identified gaps and suggestions for future work

We found several gaps and limitations in the current literature. While many studies used Kinect skeleton tracking algorithms, Yu et al. highlighted concerns regarding Kinect’s accuracy and robustness.^44^ Recent advancements in deep learning, in particular vision transformers, have given rise to more accurate and efficient methods for tracking 2D and 3D human body pose estimation.^94–100^ These methods show state-of-the-art performance on various benchmark datasets. Some also provide lightweight variants, making them potentially suitable for real-time applications. Future research should focus on applying such state-of-the-art pose estimation methods in different tele-rehab components to ensure higher precision and reliability in measuring body movements.

Secondly, the heterogeneity in performance metrics reported across studies poses a considerable challenge in comparing the performance of different methods in tele-rehab systems. For example, studies that estimate the RoMs reported different performance metrics such as RMSE, MAE, MedAE, Spearman’s R, Pearson’s R, or R-squared. Studies focusing on classification tasks reported either accuracy, F1-Score, precision, recall, or a combination of those, making direct comparisons challenging. It is recommended that researchers report all relevant performance metrics according to their study to facilitate meaningful comparisons between different methods and systems.

Thirdly, the predominant use of k-Fold or hold-out validation methods in reviewed studies may not adequately represent the real-world scenario. In tele-rehab, where patient data is diverse and could vary significantly between individuals, a cross-subject-based validation approach is more appropriate. Cross-validation techniques such as LOSO or LMSO can better mimic the challenges faced in real-life scenarios, where patients have varying abilities and characteristics. Researchers should adopt these cross-subject-based validation methods to ensure that their developments are robust and effective across different user profiles.

Moreover, we observed that studies mostly evaluated their proposed systems primarily with healthy participants, despite their intended use for patients or older adults. While initial testing with healthy individuals is valuable, it does not capture the complexities and challenges of working with patients, or older adults who have various physical conditions and RoM limitations arose from experiencing pain. This may result in overly optimistic reported performance metrics, which may not accurately reflect the performance of the system in a real-world scenario. Therefore, validating such systems with data from the intended target population is essential to ensure that the technology is effective, safe, and applicable in a clinical setting. The target population is intended to be older adults, or patients who have limited RoM across their body joints.

Additionally, many studies relied on a restricted set of rehabilitation exercises for their evaluations, often using available online datasets. However, these datasets have limitations, particularly in capturing the complexity and diversity of lower limb movements. For instance, the IRDS^86^ primarily focuses on upper body exercises like arm raises and trunk rotations, with limited coverage of lower limb exercises. Similarly, the UI-PRMD^88^ and KIMORE^87^ datasets predominantly include exercises performed in a standing or seated position, such as squats, lunges, and sit-to-stand movements. Crucially, these datasets lack comprehensive data related to lower limb exercises involving movements while lying down—a critical component of lower body rehabilitation, especially for patients with mobility impairments. Exercises like leg raises, knee bends, and ankle rotations, performed in a supine or prone position, are underrepresented in these datasets. Therefore, there is a need for the development of more comprehensive datasets that specifically focus on lower limb exercises involving movements in the knee, hip, and ankle joints. Such datasets would better support the evaluation and improvement of tele-rehab systems tailored for lower limb rehabilitation.

Lastly, a common practice in exercise assessment studies is instructing healthy participants to intentionally perform incorrect movements to generate datasets that include both correctly and incorrectly performed exercises. While this approach is practical for data collection, it may not fully capture the natural variation and challenges associated with patients or older adults who may unintentionally perform incorrect movements due to their physical conditions. Overcoming this gap requires exploring methods to collect genuine, naturally occurring incorrect movement data from the target population while addressing the inherent challenges of working with patients and older adults. For instance, by presenting participants with videos of experts performing rehabilitation exercises and instructing them to mimic the actions without providing feedback, a natural setting for the occurrence of incorrect movements could be established.

## Conclusions

This paper provides a review on the performance of recent methodologies applied in lower-limb tele-rehab platforms. The reviewed studies were classified into three technology categories: vision-based, wearable, and pressure-sensing technologies. Within each of these categories, three main modules for creating a successful automatic tele-rehab platform were discussed: joint angle measurement/estimation, activity recognition, and exercise assessment. Various approaches were identified, compared, and discussed, and several gaps and limitations in the current literature were identified. These include the heterogeneity in reported performance metrics and evaluation methods, the need for cross-subject-based validation, limited testing with the target patient population, and limited sets of rehabilitation exercises in evaluations. In addition, there is a notable call for the development of more comprehensive datasets focusing on lower limb exercises, particularly those involving movements while lying down. Future research in tele-rehab should focus on addressing these gaps to ensure its efficacy and practicality in real-world clinical settings.

## References

[1] Alexander M . Telerehabilitation: principles and practice. Philadelphia, PA: Elsevier, 2022.

[2] Osh Wiki . Musculoskeletal lower limb disorders. Washington, DC: Osh Wiki, https://oshwiki.osha.europa.eu/en/themes/musculoskeletal-lower-limb-disorders (2023, accessed 20 August 2023).

[3] Palestra G Rebiai M Courtial E , et al. Evaluation of a rehabilitation system for the elderly in a day care center. Information 2018; 10: 3.

[4] United Nations Publications . World population ageing. New York, NY: United Nations Publications, 2015.

[5] Kawamoto ALS Da Silva FSC . Depth-sensor applications for the elderly: a viable option to promote a better quality of life. IEEE Consumer Electron Mag 2018; 7: 47–56.

[6] Statistics Canada . Table 17-10-0005-01 - Population estimates on July 1, by age and gender. Ottawa, ON: Statistics, 2024. https://www150.statcan.gc.ca/t1/tbl1/en/tv.action?pid=1710000501.

[7] Statistics Canada . Projected population, by projection scenario, age and sex, 2018. as of July 1. Ottawa, ON: Statistics Canada, 2018.

[8] Kopec JA Cibere J Sayre EC , et al. Descriptive epidemiology of musculoskeletal disorders in Canada: data from the global burden of disease study. Osteoarthritis Cartilage 2019; 27: S259, https://www.sciencedirect.com/science/article/pii/S1063458419306727

[9] Safiri S Kolahi A-A Cross M , et al. Prevalence, deaths, and disability-adjusted life years due to musculoskeletal disorders for 195 countries and territories 1990–2017. Arthritis Rheumatol 2021; 73: 702–714. 33150702 10.1002/art.41571

[10] Davis PJ Cladis FP Motoyama EK (eds) Smith’s anesthesia for infants and children. 8th ed. Philadelphia, PA: Mosby, 2011.

[11] Statistics Canada . Deaths, by cause, Chapter XIII: diseases of the musculoskeletal system and connective tissue (M00 to M99). Ottawa, ON: Statistics Canada, 2018.

[12] Markus HS Brainin M . COVID-19 and stroke-a global World Stroke Organization perspective. Int J Stroke 2020; 15: 361–364. 32310017 10.1177/1747493020923472 PMC11927026

[13] Veras M Stewart J Deonandan R , et al. Cost analysis of a home-based virtual reality rehabilitation to improve upper limb function in stroke survivors. GJHS 2020; 12: 98.

[14] Eichler S Salzwedel A Rabe S , et al. The effectiveness of telerehabilitation as a supplement to rehabilitation in patients after total knee or hip replacement: randomized controlled trial. JMIR Rehabil Assist Technol 2019; 6: e14236. 31697239 10.2196/14236 PMC6873150

[15] Chen J Sun D Zhang S , et al. Effects of home-based telerehabilitation in patients with stroke: a randomized controlled trial. Neurology 2020; 95: e2318–e2330. 32999058 10.1212/WNL.0000000000010821

[16] Junata M Cheng KC-C Man HS , et al. Kinect-based rapid movement training to improve balance recovery for stroke fall prevention: a randomized controlled trial. J NeuroEng Rehabil 2021; 18: 150. 34635141 10.1186/s12984-021-00922-3 PMC8503723

[17] Gandolfi M Munari D Geroin C , et al. Sensory integration balance training in patients with multiple sclerosis: a randomized, controlled trial. Mult Scler 2015; 21: 1453–1462. 25583852 10.1177/1352458514562438

[18] Gandolfi M Geroin C Dimitrova E , et al. Virtual reality telerehabilitation for postural instability in Parkinson’s disease: a multicenter, single-blind, randomized, controlled trial. BioMed Res Int 2017; 2017: 7962826. 29333454 10.1155/2017/7962826 PMC5733154

[19] Imam B Miller WC McLaren L , et al. Feasibility of the Nintendo WiiFit™ for improving walking in individuals with a lower limb amputation. SAGE Open Med 2013; 1: 2050312113497942. 26770676 10.1177/2050312113497942 PMC4687776

[20] Tao G Miller WC Eng JJ , et al. Group-based telerehabilitation intervention using Wii fit to improve walking in older adults with lower limb amputation (WiiNWalk): a randomized control trial. Clin Rehabil 2022; 36: 331–341. 34841917 10.1177/02692155211061222

[21] Oyama S Saeki M Kaneta S , et al. Telerehabilitation based on markerless motion capture and IMT-2020 (5G) networks. Stud Health Technol Inf 2022; 290: 1108–1109. 10.3233/SHTI220291 35673229

[22] Perez Medina JL Gonzalez M Pilco HM , et al. Usability study of a web-based platform for home motor rehabilitation. IEEE Access 2019; 7: 7932–7947.

[23] Camilo R Edwin G Andres C , et al. Addressing motivation issues in physical rehabilitation treatments using exergames. In: Serrano CJE Martínez-Santos JC (eds) Advances in computing. Cham, Switzerland: Springer International Publishing, 2018, pp. 459–470.

[24] Moffet H Saey D Coats V , et al. Reliability and usability of the eChez-soi in-home telerehabilitation platform: a new internet-based communication and real-time monitoring software solution combined with interactive exercises - results of a longitudinal pilot study in four patients with lung cancer. In: Proceedings of the 1st international conference on information and communication technologies for ageing well and e-health, Lisbon, Portugal, 20 May 2015–22 May 2015, pp. 137–142. SCITEPRESS - Science and and Technology Publications.

[25] Coats V Moffet H Vincent C , et al. Feasibility of an eight-week telerehabilitation intervention for patients with unresectable thoracic neoplasia receiving chemotherapy: a pilot study. Canadian J of Resp, Critical Care, and Sleep Med 2020; 4: 14–24.

[26] Adolf J Dolezal J Kutilek P , et al. Automatic telerehabilitation system in a home environment using computer vision. Stud Health Technol Inf 2020; 273: 142–148. 10.3233/SHTI200629 33087604

[27] Antón D Goñi A Illarramendi A . Exercise recognition for Kinect-based telerehabilitation. Methods Inf Med 2015; 54: 145–155. 25301322 10.3414/ME13-01-0109

[28] Antón D Nelson M Russell T , et al. Validation of a kinect-based telerehabilitation system with total hip replacement patients. J Telemed Telecare 2016; 22: 192–197. 26130735 10.1177/1357633X15590019

[29] Barelle C Courtial E Vellidou E , et al. Tele-monitoring and diagnostic for fall prevention: the KINOPTIM concept. In: 2014 IEEE-EMBS international conference on biomedical and health informatics (BHI), Valencia, Spain, 1–4 June 2014.

[30] Barriga A Conejero JM Hernández J , et al. A vision-based approach for building telecare and telerehabilitation services. Sensors 2016; 16. 10.3390/s16101724 PMC5087511 27763540

[31] Barzegar Khanghah A Fernie G Roshan Fekr A . Design and validation of vision-based exercise biofeedback for tele-rehabilitation. Sensors 2023; 23: 1206. 36772246 10.3390/s23031206 PMC9920527

[32] Bijalwan V Semwal VB Singh G , et al. Heterogeneous computing model for post‐injury walking pattern restoration and postural stability rehabilitation exercise recognition. Expet Syst 2022; 39(6): e12706.

[33] Chowdhury SH Amin MA Rahman AKMM , et al. Assessment of rehabilitation exercises from depth sensor data. In: 2021 24th international conference on computer and information technology (ICCIT), Dhaka, Bangladesh, 18–20 December 2021, pp. 1–7. IEEE.

[34] Deb S Islam MF Rahman S , et al. Graph convolutional networks for assessment of physical rehabilitation exercises. IEEE Trans Neural Syst Rehabil Eng 2022; 30: 410–419. 35139022 10.1109/TNSRE.2022.3150392

[35] Decroos T Schütte K Beéck T , et al. AMIE: automatic monitoring of indoor exercises. In: Brefeld U Curry E Daly E , et al. (eds) Machine learning and knowledge discovery in databases. Cham, Switzerland: Springer International Publishing, 2019, pp. 424–439.

[36] Guo Q Khan S . Exercise-specific feature extraction approach for assessing physical rehabilitation. In: 4th IJCAI workshop on AI for aging, rehabilitation and intelligent assisted living, Montreal, QC, June 2021.

[37] Kanade A Sharma M Muniyandi M . Tele-EvalNet: a low-cost, teleconsultation system for home based rehabilitation of stroke survivors using multiscale CNN-ConvLSTM architecture. In: Computer Vision - ECCV 2022 Workshops. Cham, Switzerland: Springer, 2023, pp. 738–750.

[38] Kouris I Tsirbas C Tagaris T , et al. KINOPTIM: the medical business intelligence module for fall prevention of the elderly. In: 2015 IEEE 15th international conference on bioinformatics and bioengineering (BIBE), Belgrade, Serbia, 2–4 November 2015, pp. 1–4. IEEE.

[39] Reby K Dulau I Dubrasquet G , et al. Graph transformer for physical rehabilitation evaluation. In: 2023 IEEE 17th international conference on automatic face and gesture recognition (FG), Waikoloa Beach, HI, 5–8 January 2023, pp. 1–8. IEEE.

[40] Rosique F Losilla F Navarro PJ . Applying vision-based pose estimation in a telerehabilitation application. Appl Sci 2021; 11: 9132.

[41] Rybarczyk Y Pérez Medina JL Leconte L , et al. Implementation and assessment of an intelligent motor tele-rehabilitation platform. Electronics 2019; 8: 58.

[42] Rybarczyk Y Deters JK Gonzalvo AA , et al. ePHoRt project: a web-based platform for home motor rehabilitation. In: Rocha Á Correia AM Adeli H , et al. (eds) Recent advances in information systems and technologies. Cham, Switzerland: Springer International Publishing, 2017, pp. 609–618.

[43] Ye M Yang C Stankovic V , et al. A depth camera motion analysis framework for tele-rehabilitation: motion capture and person-centric kinematics analysis. IEEE J Sel Top Signal Process 2016; 10: 877–887.

[44] Yu K Barmaki R Unberath M , et al. On the accuracy of low-cost motion capture systems for range of motion measurements (conference presentation). In: Medical imaging 2018: imaging informatics for healthcare, research, and applications, Houston, TX, 13–15 February 2018, p. 31. SPIE.

[45] Zhao W Lun R Espy DD , et al. Rule based realtime motion assessment for rehabilitation exercises. In: 2014 IEEE symposium on computational intelligence in healthcare and e-health (CICARE 2014), Orlando, FL, 9–12 December 2014, pp. 133–140. IEEE.

[46] Zhao W Reinthal MA Espy DD , et al. Rule-based human motion tracking for rehabilitation exercises: realtime assessment, feedback, and guidance. IEEE Access 2017; 5: 21382–21394.

[47] Zheng K Wu J Zhang J , et al. A skeleton-based rehabilitation exercise assessment system with rotation invariance. IEEE Trans Neural Syst Rehabil Eng 2023; 31: 2612–2621. 37276100 10.1109/TNSRE.2023.3282675

[48] Khanghah AB Fernie G Fekr AR . Joint angle estimation during shoulder abduction exercise using contactless technology. Biomed Eng 2023; 23(1): 11. DOI: 10.1186/s12938-024-01203-5. PMC10822169 38281988

[49] Barzegar Khanghah A Fernie G Roshan Fekr A . A novel approach to tele-rehabilitation: implementing a biofeedback system using machine learning algorithms. Mach Learn Appl 2023; 14: 100499, https://www.sciencedirect.com/science/article/pii/s266682702300052x

[50] Davarzani S Saucier D Talegaonkar P , et al. Closing the wearable gap: foot–ankle kinematic modeling via deep learning models based on a smart sock wearable. Wearable Technol 2023; 4: e4. 38487777 10.1017/wtc.2023.3 PMC10936318

[51] Wood DS Jensen K Crane A , et al. Accurate prediction of knee angles during open-chain rehabilitation exercises using a wearable array of nanocomposite stretch sensors. Sensors 2022; 22: 2499. 35408112 10.3390/s22072499 PMC9003122

[52] Haladjian J Bredies K Brugge B . KneeHapp textile: a smart textile system for rehabilitation of knee injuries. In: 2018 IEEE 15th international conference on wearable and implantable body sensor networks (BSN), Las Vegas, NV, 4–7 March 2018, pp. 9–12. IEEE.

[53] Nakamoto H Yamaji T Hirata I , et al. Joint angle measurement by stretchable strain sensor. J Ambient Intell Hum Comput 2018; 14: 14623–14628.

[54] Song Q Ma X Liu Y . Continuous online prediction of lower limb joints angles based on sEMG signals by deep learning approach. Comput Biol Med 2023; 163: 107124. 37315381 10.1016/j.compbiomed.2023.107124

[55] Wang C Li X Guo Y , et al. Classification of human movements with and without spinal orthosis based on surface electromyogram signals. Medicine in Novel Techn and Dev 2022; 16: 100165, https://www.sciencedirect.com/science/article/pii/S2590093522000522

[56] Marin-Pardo O Phanord C Donnelly MR , et al. Development of a low-cost, modular muscle-computer interface for at-home telerehabilitation for chronic stroke. Sensors 2021; 21: 1806, https://www.ncbi.nlm.nih.gov/pmc/articles/PMC7961888/33807691 10.3390/s21051806 PMC7961888

[57] Yassin MM Saber AM Saad MN , et al. Developing a low-cost, smart, handheld electromyography biofeedback system for telerehabilitation with clinical evaluation. Medicine in Novel Technology and Devices 2021; 10: 100056, https://www.sciencedirect.com/science/article/pii/S2590093520300308

[58] Wang J Wang L Xi X , et al. Estimation and correlation analysis of lower limb joint angles based on surface electromyography. Electronics 2020; 9: 556.

[59] Nissa R Karmakar NC Baghini MS . Embedded machine learning on accelerometer data for exercise classification. In: 2023 IEEE applied sensing conference (APSCON), Bengaluru, India, 22–24 January 2023, pp. 1–3. IEEE.

[60] García-de-Villa S Casillas-Pérez D Jiménez-Martín A , et al. Simultaneous exercise recognition and evaluation in prescribed routines: approach to virtual coaches. Expert Syst Appl 2022; 199: 116990, https://www.sciencedirect.com/science/article/pii/S0957417422004110

[61] Kim J-K Bae M-N Lee KB , et al. Identification of patients with sarcopenia using gait parameters based on inertial sensors. Sensors 2021; 21: 1786, https://www.mdpi.com/1424-8220/21/5/1786 33806525 10.3390/s21051786 PMC7961754

[62] Lai Y-C Kan Y-C Lin Y-C , et al. AIoT-enabled rehabilitation recognition system-exemplified by Hybrid lower-limb exercises. Sensors 2021; 21: 4761. 34300501 10.3390/s21144761 PMC8309886

[63] Majumder S Deen MJ . Wearable IMU-based system for real-time monitoring of lower-limb joints. IEEE Sensor J 2021; 21: 8267–8275.

[64] Abdollahi M Ashouri S Abedi M , et al. Using a motion sensor to categorize nonspecific low back pain patients: a machine learning approach. Sensors 2020; 20(12): 3600. 32604794 10.3390/s20123600 PMC7348921

[65] Bevilacqua A Ciampi G Argent R , et al. Combining real-time segmentation and classification of rehabilitation exercises with LSTM networks and pointwise boosting. AAAI 2020; 34: 13229–13234.

[66] Katzan IL Thompson NR George SZ , et al. The use of STarT back screening tool to predict functional disability outcomes in patients receiving physical therapy for low back pain. Spine J 2019; 19: 645–654. 30308254 10.1016/j.spinee.2018.10.002 PMC7341439

[67] Argent R Drummond S Remus A , et al. Evaluating the use of machine learning in the assessment of joint angle using a single inertial sensor. J Rehabil Assist Technol Eng 2019; 6: 2055668319868544. 31452927 10.1177/2055668319868544 PMC6700879

[68] Bevilacqua A Huang B Argent R , et al. Automatic classification of knee rehabilitation exercises using a single inertial sensor: a case study. In: 2018 IEEE 15th international conference on wearable and implantable body sensor networks (BSN), Las Vegas, NV, 4–7 March 2018, pp. 21–24.

[69] Pereira A Folgado D Nunes F , et al. Using inertial sensors to evaluate exercise correctness in electromyography-based home rehabilitation systems. In: 2019 IEEE international symposium on medical measurements and applications (MeMeA), Istanbul, Turkey, 26–28 June 2019, pp. 1–6. IEEE.

[70] Bennett T Kumar P Garate VR . A machine learning model for predicting sit-to-stand trajectories of people with and without stroke: towards adaptive robotic assistance. Sensors 2022; 22: 4789. 35808285 10.3390/s22134789 PMC9269285

[71] Choffin Z Jeong N Callihan M , et al. Lower body joint angle prediction using machine learning and applied biomechanical inverse dynamics. Sensors 2022; 23: 228. 36616825 10.3390/s23010228 PMC9824079

[72] Wang A Li D Fan N , et al. Piezoresistive-based gait monitoring technique for the recognition of knee osteoarthritis patients. IEEE Access 2022; 10: 123874–123884.

[73] Wicaksono I Hwang PG Droubi S , et al. 3DKnITS: three-dimensional digital knitting of intelligent textile sensor for activity recognition and biomechanical monitoring. Annu Int Conf IEEE Eng Med Biol Soc 2022; 2022: 2403–2409. 36086308 10.1109/EMBC48229.2022.9871651

[74] Wijekoon A Wiratunga N Cooper K . Heterogeneous multi-modal sensor fusion with Hybrid attention for exercise recognition. In: 2020 international joint conference on neural networks (IJCNN), Glasgow, UK, 19–24 July 2020, pp. 1–8. IEEE.

[75] Martini E Fiumalbi T Dell’Agnello F , et al. Pressure-sensitive insoles for real-time gait-related applications. Sensors 2020; 20(5): 1448, https://www.ncbi.nlm.nih.gov/pmc/articles/PMC7085512/32155828 10.3390/s20051448 PMC7085512

[76] Wijekoon A Wiratunga N Cooper K . MEx: multi-modal exercises dataset for human activity recognition. 2019. arXiv:1908.08992.

[77] Lee S-S Choi ST Choi S-I . Classification of gait type based on deep learning using various sensors with smart insole. Sensors 2019; 19(8): 1757. 31013773 10.3390/s19081757 PMC6514988

[78] Bayan S Assaf K Yassin M , et al. Inexpensive virtual assisted rehabilitation system (VARS) for lower part injuries. In: 2017 fourth international conference on advances in biomedical engineering (ICABME), Beirut, Lebanon, 18–20 October 2017, pp. 1–4. IEEE.

[79] Jagos H Pils K Haller M , et al. Mobile gait analysis via eSHOEs instrumented shoe insoles: a pilot study for validation against the gold standard GAITRite®. J Med Eng Technol 2017; 41: 375–386. 28573909 10.1080/03091902.2017.1320434

[80] Sun Q Gonzalez E Sun Y . On bed posture recognition with pressure sensor array system. In: 2016 IEEE sensors, Orlando, FL, 30 October 2016–03 November 2016, pp. 1–3: IEEE.

[81] Huang M-C Liu JJ Xu W , et al. Using pressure map sequences for recognition of on bed rehabilitation exercises. IEEE J Biomed Health Inform 2014; 18: 411–418. 24608046 10.1109/JBHI.2013.2296891

[82] OpenNI 2 SDK Binaries & Docs . OpenNI user guide, https://www.openni.org/documentation

[83] Cao Z Simon T Wei S-E , et al. Realtime multi-person 2D pose estimation using part affinity fields. In: 2017 IEEE conference on computer vision and pattern recognition (CVPR), Honolulu, HI, 21–26 July 2017, pp. 1302–1310. IEEE.

[84] VICON motion capture system, https://www.vicon.com/motion-capture

[85] Polaris vicra optical tracking system, https://www.ndigital.com/optical-measurement-technology/polaris-vicra/

[86] Miron A Sadawi N Ismail W , et al. Intellirehabds (Irds)—a dataset of physical rehabilitation movements. Data 2021; 6: 1–13.

[87] Capecci M Ceravolo MG Ferracuti F , et al. The KIMORE dataset: kinematic assessment of movement and clinical scores for remote monitoring of physical REhabilitation. IEEE Trans Neural Syst Rehabil Eng 2019; 27: 1436–1448. 31217121 10.1109/TNSRE.2019.2923060

[88] Vakanski A Jun HP Paul D , et al. A data set of human body movements for physical rehabilitation exercises. Data 2018; 3: 2. 29354641 10.3390/data3010002 PMC5773117

[89] Biopac Systems I . Upgrade to MP36 system - windows OS | MP36U-W, MP36U-M|education|BIOPAC, https://www.biopac.com/product/upgrade-to-mp36-system/

[90] Ockendon M Gilbert RE . Validation of a novel smartphone accelerometer-based knee goniometer. J Knee Surg 2012; 25: 341–345. 23150162 10.1055/s-0031-1299669

[91] Ma J . Innovative intelligent sensors to objectively understand exercise interventions for older adults. Doctoral Thesis. Loughborough University, Loughborough, UK, 2019.

[92] Zhao W Espy DD Reinthal A . A validation study of rehabilitation exercise monitoring using Kinect. In: Khosrow-Pour M (ed) Advanced methodologies and technologies in medicine and healthcare. Hershey PA: IGI Global, 2019, pp. 466–482.

[93] Ettefagh A Roshan Fekr A . Enhancing automated lower limb rehabilitation exercise task recognition through multi-sensor data fusion in tele-rehabilitation. BioMedical Engineering Online. 2024; 23(1). DOI: 10.1186/s12938-024-01228-w. PMC10949721 38504279

[94] Bazarevsky V Grishchenko I Raveendran K , et al. BlazePose: on-device real-time body pose tracking. 2020. arXiv:2006.10204.

[95] Güler RA Neverova N Kokkinos I . DensePose: dense human pose estimation in the wild. 2018. arXiv:1802.00434.

[96] Liu H Liu F Fan X , et al. Polarized self-attention: towards high-quality pixel-wise regression. 2021. arXiv:2107.00782.

[97] Pavllo D Feichtenhofer C Grangier D , et al. 3D human pose estimation in video with temporal convolutions and semi-supervised training. 2018. arXiv:1811.11742.

[98] Xu Y Zhang J Zhang Q , et al. ViTPose: simple vision transformer baselines for human pose estimation. 2022. arXiv:2204.12484.

[99] Yan S Xiong Y Lin D . Spatial temporal graph convolutional networks for skeleton-based action recognition. 2018. arXiv:1801.07455.

[100] Zhou X Huang Q Sun X , et al. Towards 3D human pose estimation in the wild: a weakly-supervised approach. In: 2017 IEEE international conference on computer vision (ICCV), Venice, Italy, 22–29 October 2017.

